# Morphological changes of lenticels and their role in gas exchange and sprouting physiology of potato tubers during postharvest storage

**DOI:** 10.3389/fpls.2025.1595828

**Published:** 2025-06-11

**Authors:** Lembe S. Magwaza, Antonio J. B. Bernal, Gemma A. Chope, M. Carmen Alamar, Leon A. Terry

**Affiliations:** ^1^ Postharvest Research Group, Cranfield University, Bedfordshire, United Kingdom; ^2^ PepsiCo International Limited, Beaumont Park, Leicester, United Kingdom

**Keywords:** bud development, sprout suppressant, ethylene, 1-MCP, image analysis, respiration rate

## Abstract

The application of exogenous gases has been used to suppress sprouting in stored potato tubers. However, their efficacy in extending ecodormancy largely depends on achieving optimal gas exchange between the storage atmosphere and the tuber itself. This study aimed to investigate morphological variations and spatial distribution of lenticels and apical buds and to identify their potential role in tuber respiration rate and sprouting of five potato cultivars (‘Hermes’, ‘Lady Claire’, ‘Lady Rosetta’, ‘Saturna’, and ‘VR808’) during storage. Results revealed a consistent spatial pattern wherein the apical section of potato tubers exhibited significantly higher bud counts compared to lateral and stolon regions. ‘Lady Claire’ stood out as having the highest number of apical buds among the cultivars studied. Digital image analysis showed a seven times higher number of buds surrounding the apical eye and these were generally smaller than those distributed across the skin. ‘Saturna’ displayed double the lenticel density (12 lenticels cm^-2^) in smaller tubers, suggesting an inverse relationship between tuber size and lenticel density. ‘Lady Claire’ and ‘Saturna’ had respiration rates of 2.75 and 1.9 mL CO_2_ kg^-1^ h^-1^, respectively, and were selected for additional respiration and ethylene efflux analyses. In both cultivars, distinct spatial differences were observed, with the apical section exhibiting a seven-fold increase in lenticel density compared to the lateral and stolon sections. Respiration rate increased five-fold when apical lenticels were blocked, whereas it decreased 30-fold when the apical was the only unblocked section, suggesting differential physiological activity across lenticel locations. The apical sections, with the highest lenticel density, exhibited elevated respiration rates as a stress-induced physiological response upon blockage, compared to the lateral and stolon sections. Lenticels changed their morphology during storage, erupting before bud movement, suggesting lenticel eruption could be used as a pre-symptomatic visual marker of dormancy break. This study highlights the critical role that lenticel morphology and spatial distribution may have in determining potato tuber gas exchange and refining allied storage regimes.

## 1 Introduction

Potato tubers are typically cured under cool, dark and ventilated conditions for 7–10 days after harvest to thicken their skin through suberization. Curing also reduces the respiration rate of potato tubers during postharvest storage, thereby, potentially extending dormancy (Verdaguer et al., 2016; Tosetti et al., 2021; Bethke, 2023). Storage life can be prolonged further by keeping tubers under cold temperatures (3.5 °C for fresh and 8.5 °C for processing – to extend eco-dormancy) and applying sprout suppressants (Terry et al., 2013; Alamar et al., 2017; Sharma et al., 2021). Among the latter, chlorpropham isopropyl-N-(3-chlorophenyl carbamate (CIPC), valued for its long-term sprout inhibition with just a single application, has been used for more than 60 years (Pinhero and Yada, 2016; Sharma et al., 2021). However, due to concerns about its toxicity, the European Union withdrew its authorization in 2019 (EUCRI, 2019). While CIPC is still used in many countries, this has spurred interest in developing alternative residue-free postharvest treatments for sprout suppression (Alamar et al., 2017; Sharma et al., 2021).

Exogenous ethylene application has not only been commercialized as an alternative sprout suppressant but has received significant research attention (Daniels-Lake et al., 2005, 2013; Foukaraki et al., 2012, 2014, Foukaraki et al., 2016a, b; Alamar et al., 2017; Tosetti et al., 2021). Despite its proven efficacy in terms of sprout suppression, continuous ethylene treatment has been associated with increased tuber respiration rates and induced sucrose hydrolysis, resulting in the accumulation of reducing sugars, a phenomenon known as ethylene-induced sweetening (Daniels-Lake et al., 2005; Foukaraki et al., 2012, 2016). Ethylene-induced accumulation of reducing sugars can be mitigated through a physiologically targeted approach, wherein a 24-hour pretreatment with 1-methylcyclopropene (1-MCP) (1 μL L^-1^) is applied prior to ethylene exposure (10 μL L^-1^) (Prange et al., 2005; Daniels-Lake et al., 2008; Foukaraki et al., 2014, 2016; Tosetti et al., 2021).

Recent studies in low ethylene-producing crops, including onions (Põldma et al., 2012; Cools et al., 2014; Alamar et al., 2020), sweetpotato (Imahori et al., 2007; Amoah et al., 2016, 2017), and potatoes (Foukaraki et al., 2016a; Alamar et al., 2017; Dako et al., 2021), have shown that controlled atmosphere (CA) storage, achieved by reducing O_2_ and increasing CO_2_ partial pressures, is an effective method for slowing down respiration and senescence while minimizing the adverse effects of exogenous ethylene (Amoah et al., 2017; Kiaitsi et al., 2020). Therefore, CA storage could serve as an effective alternative and/or additive treatment to ethylene, effectively extending the storage life of potatoes with minimal adverse effects. Collectively, the literature above indicates that the most effective alternative sprout suppressants involve gaseous postharvest treatments (*i.e*., ethylene, CO_2,_ and O_2_).

In potato tubers, O_2_ and CO_2_ exchange occurs through lenticels, which are naturally occurring openings (without guard cells), initially made up of radial files of rounded, undifferentiated cells (Bethke, 2023). A study comparing gaseous diffusivity between the skin and lenticels reported a 300 times lower CO_2_ diffusion (6.57x10^−7^ cm s^−1^) compared to the value observed for lenticels (2.00 x 10^−4^ cm s^−1^) (Burton, 1965; Burton and Wigginton, 1970; Abdul-Baki and Solomos, 1994), indicating that lenticels are the primary route for gaseous exchange. A positive correlation between the number of open lenticels and peel permeability, leading to increased gaseous exchange, has been reported on apples (Kritzinger and Lötze, 2019; Khanal et al., 2020) and pomegranate fruit (Lufu et al., 2021). However, there are no similar detailed studies on lenticel structures of tuberous storage organs. We hypothesize that the morphology and number of open lenticels might similarly influence gas permeability, with variations depending on lenticel size, cultivar, and storage conditions. While the hypothesis is that postharvest gaseous exchange in potato tubers predominantly occurs through lenticels, there remains a gap in our understanding regarding the role of lenticels, morphological characteristics, spatial distribution across the tuber, and their physiological variation during storage and dormancy break. Such research is warranted to elucidate the mechanisms underlying respiration and dormancy regulation in potato tubers. Therefore, the current study was conducted to characterize the morphological changes in lenticels during storage and to gain insight into the role of lenticel position and number in mediating the gas exchange of potato tubers. The five studied potato cultivars, ‘Hermes’, ‘Lady Claire’, ‘Saturna’, ‘VR 808’, and ‘Lady Rosetta’, are commonly used on crisp (potato chip) production, with the first four preferred for long-term storage due to their high dormancy and low reducing sugar content, while ‘Lady Rosetta’ is more suitable for fresh processing or short-term storage.

## 2 Materials and methods

### 2.1 Plant material and experimental design

The experiments were carried out using five potato (*Solanum tuberosum* L.) cultivars, namely, ‘Hermes’, ‘Lady Claire’, ‘Lady Rosetta’, ‘Saturna’, and ‘VR808’, provided by PepsiCo International Ltd. The tubers were harvested on the 19^th^ of September 2013 for Shropshire (52°42’2.4” N; 2°36’54.3” W), the 23^rd^ of September for Yorkshire (54°10’13.9” N; 0°39’56.1” W), and the 7^th^ of October 2013 for Norfolk (52°51’24.4” N; 0°49’24.8” E). Following commercial practice, tubers were transferred to Sutton Bridge Crop Storage Research (Agriculture and Horticulture Development Board, Sutton Bridge, Lincolnshire, UK) for curing. After curing, tubers were transported within 2 h to Cranfield University, UK, where temperature was gradually reduced by 0.3 °C per day until reaching the final storage temperature of 10 °C. CIPC treatments were managed in Sutton Bridge, where standard commercial practice involving three applications was followed. The treated tubers were then placed in trays and stored in the cold room at 10 °C for five months.

### 2.2 Characterization of tuber periderm: density of buds and lenticels and their spatial distribution

Tubers of the cultivars ‘Hermes’, ‘Saturna’, ‘Lady Claire’ and ‘VR808’ exhibited the same shape were classified into four groups based on their size: small, *x* < 80 cm^2^; medium, 80 cm^2^ < *x* < 120 cm^2^; large, 120 cm^2^ < *x* < 160 cm^2^; very large, *x* > 160 cm^2^ (**Supplementary Table 1**). Overall tubers from the ‘Hermes’ cultivar were *circa* 1.4 times larger than the rest of the cultivars.

Six replicates of six tubers per replicate in each size group and cultivar were rinsed with tap water at room temperature, *circa* 18-20 °C, and then submerged in a 1.5 g L^-1^ methylene blue dye solution for 15 min. Potatoes were placed in a desiccator and exposed to vacuum infiltration for 30 min before being gently scrubbed with a wet sponge to remove excess dye until a pattern of blue dots appeared on the skin - confirming dye diffusion through lenticels. Tubers were then peeled (*circa* 2 mm thick), and the skins of the stolon, lateral, and apical sections were placed on a white paper next to a ruler (**Supplementary Figure 1**). Skin surface area, as well as lenticel and bud distribution and size, were calculated using the free version of ImageJ image-processing software (ImageJ, U.S. National Institutes of Health, Bethesda, Maryland, USA) (Schneider et al., 2012).

The procedure for counting lenticels followed the “Automatic Particle Counting” method outlined on the ImageJ website, while buds were counted and validated using the “Manual Counting” tool due to their low numbers. Lenticel counts were also cross-validated with manual counting when needed. The “Automatic Particle Counting” process began by converting the original image into an 8-bit binary format. A threshold was set, converting the objects of interest (lenticels) into white pixels on a black background (**Supplementary Figure 2A**) (Sezgin and Sankur, 2004). The threshold image was then transformed into a second 8-bit image for watershed separation, generating an Euclidian distance map where central pixels remained white while those close to black dots became progressively darker. Centres of objects were calculated, and the white points were dilated until they touched neighbouring white pixels, marking the watershed boundaries (**Supplementary Figure 2B**). Segmented particles of images were analyzed using the “Analyse Particles” tool, allowing exclusion based on maximum/minimum size and roundness value (0.0 to 1.0).

### 2.3 Respiration rate measurements

To investigate the role of each tuber section in gas exchange, selected ‘Saturna’ and ‘Lady Claire’ tubers from Norfolk and Shropshire fields underwent one of eight obstruction treatments. Six tubers per cultivar had specific tuber sections (stolon, lateral, and apical) obstructed in eight patterns to limit gas diffusion through lenticels, as detailed in **Supplementary Figure 3**. For each pattern, lenticels were coated with Araldite resin, applied with a micropipette tip, and allowed to air dry for 5 min. Treatments included blocking individual sections (3 patterns), two-section combinations (3 patterns), full obstruction, and a control with no obstruction. Once cured, the coated sections were fully covered with a combination of epoxy resin (Araldite^®^, CY212, Agar Scientific, Rotherham, UK) and food-grade white grease (Calcium Sulfonate, Premier Fuels & Lubricants Ltd, Staffordshire, UK) and respiration rates were measured in mL CO_2_ kg^-1^ h^-1^.

The respiration rate was assessed using the Sable Respirometry System (model 1.3.8 Pro, Sable System International, NV, USA.) at room temperature for individual tubers (Collings et al., 2013). For the preliminary respiration assay, six tubers of every cultivar were selected; each tuber was placed into 3 L hermetically sealed jars and continuously flushed with air to prevent the development of modified atmosphere conditions. Five tubers were collected in the same 3 L jars when the respiration was measured for the blocked tubers. Respiration rates were determined by measuring excurrent CO_2_ levels for 2 min. The baseline (empty jar) was recorded one minute before and after each sample. The tubers were then treated with ethylene (10 μL L^-1^ for 24 h); they were placed in a 100 L box sealed with water (gas-tight sealing) and supplemented with ethylene. Ethylene efflux was measured at room temperature for individual tubers using an ethylene detector (Sensor Sense B.V., Nijmegen, The Netherlands). The efflux was recorded using photoacoustic spectroscopy (EDT-300 ethylene detector), VC-1 valve control box (continuous flow 4 mL h^-1^), and CAT-1 catalyzer from Sensor Sense B.V. (Nijmegen, The Netherlands). The measurement interval of the EDT-300 was 5 s with a response time of 30 s.

### 2.4 Morphological changes in lenticels during storage

The effect of short ethylene treatment (Et) (10 μL L^-1^) on the physiology of CIPC (14 g tonne^-1^; CAS number 101-21-3, Sigma-Aldrich, UK) and non-CIPC treated tubers was assessed in the ‘Lady Rosetta’, ‘VR808’, and ‘Lady Claire’ cultivars stored at 10 °C (-CIPC/-Et, +CIPC/-Et, +CIPC/+Et, and -CIPC/+Et). Data were collected on five sampling points: 0, 17, 45, 73, and 199 days of storage. ‘Lady Rosetta’ was sampled until 98 days of storage, at which point it had sprouted.

For ethylene treatment, six tubers from each cultivar and CIPC combination (+CIPC, -CIPC) were collected 24 h before sampling, moved to sealed boxes, and treated with ethylene (10 μL L^-1^) for 24 h. Six of the non-ethylene treated tubers (–Et) were collected on the sampling day from sealed boxes flushed with air.

At each time point, the selected tubers of each treatment were washed and physically characterized as mentioned in Section 2.2, above, and the respiration rate was measured individually for every tuber. After characterization, three slices of skin (15 mm x 15 mm x 2 mm) from each section (stolon, lateral and apical) were randomly collected with a scalpel; the lenticels on each skin section were viewed using an optical stereo microscope (Nikon, SMZ-1, Tokyo, Japan). Lenticels were classified as erupted or non-erupted (**Supplementary Figure 4**); the percentage of erupted lenticels was calculated per cm^2^ for every tuber section.

Skin and lenticels of six ‘VR808’ tuber replicates were analyzed using the environmental scanning electron microscope (ESEM; FEI XL30 ESEM, Philips, The Netherlands) with the low-vacuum chamber pressure set 0.8 Torr. Small portions (15 mm x 15 mm x 2 mm) of skin from three sections (stolon, lateral, and apical) were randomly selected. Samples were cut and placed in a sample carousel for analysis. Six lenticels from each section were imaged, and the images were processed using EDS Software (Oxford Instruments, UK). Each surface area image focused on one lenticel and the surrounding skin tissue, with resolutions ranging from 1 mm to 100 µm. Atomic composition spectra from both outside and inside the lenticel were obtained with an X-ray analyzer. The software enabled the mapping of the atomic composition of the entire image or the analysis of a selected area.

### 2.5 Statistical analyses

Statistical analysis was carried out using STATISTICA for Windows 12^th^ Edition (StatSoft Tulsa, OK 74104, USA). A common baseline was used to compare with other treatments. Analysis of variance (ANOVA) was performed to analyze the differences among group means. The independence of observations was assumed since the experimental designs were completely randomized. Analyses of residuals were carried out to identify the distribution of data. Skewed data were normalized using log, square root, or Arcsin conversions, where needed. The *post hoc* Fisher test was carried out to obtain the least significant difference (LSD) using critical values of *t* (*p* ≤ 0.05).

## 3 Results and discussion

### 3.1 Spatial distribution of buds and lenticels along tubers

**Figure 1:**
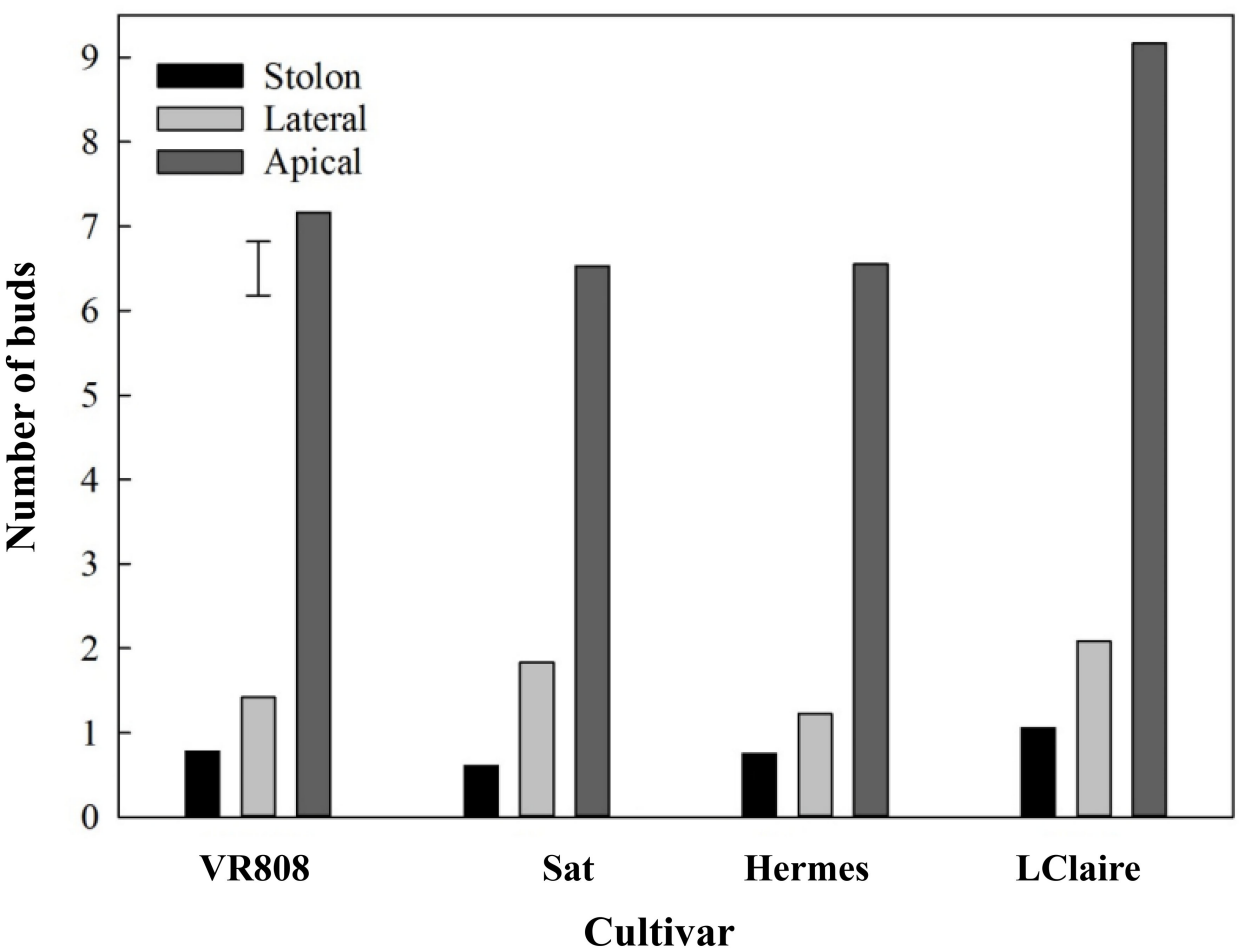
Spatial distribution of buds per section along tubers (n = 36) of four cultivars, namely, ‘VR808’, ‘Saturna’ (Sat), ‘Hermes’, and ‘Lady Claire’ (LClaire). The error bar represents the least significant difference (LSD_0.05_) for the interaction cultivar*spatial position, which was 0.6.

The spatial distribution of lenticels and buds showed a consistent pattern across all cultivars studied. The number of buds was seven times higher in the apical section than in the lateral and stolon (**Figure 1**). ‘Lady Claire’, in particular, showed the overall highest number of buds (average value of nine) in the apical section compared to the other cultivars with an average value of six.

**Figure 2:**
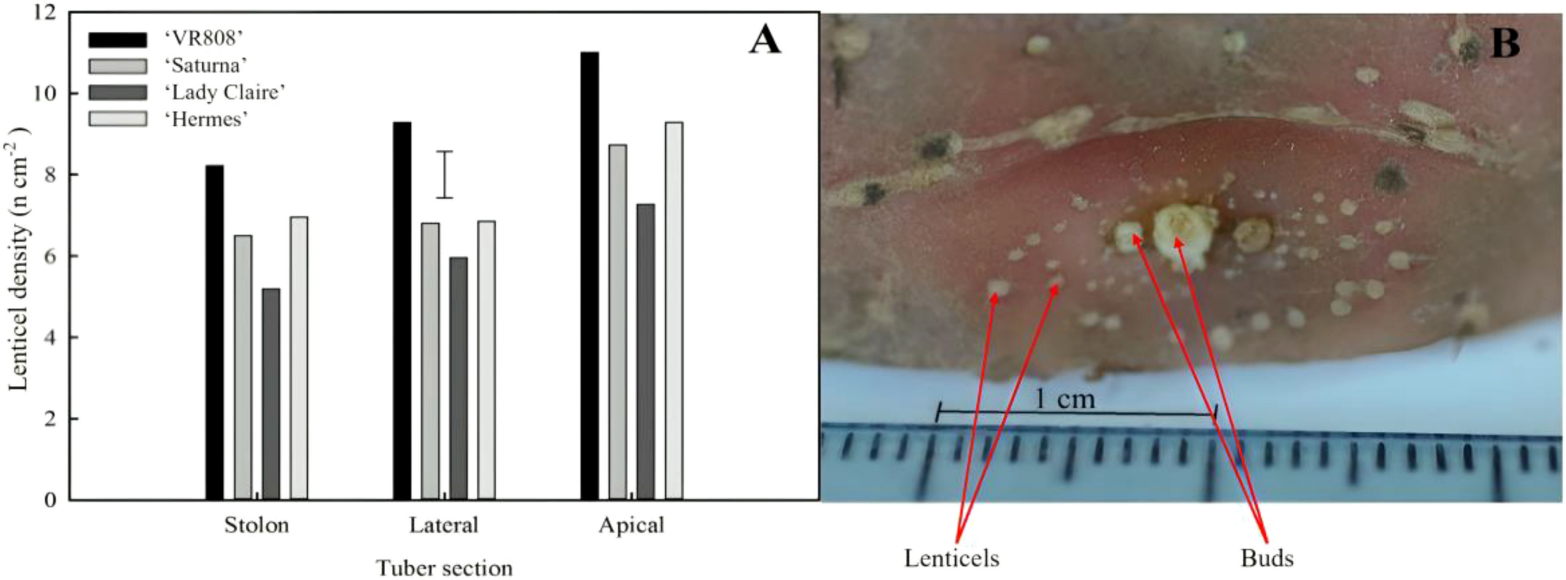
Spatial distribution of lenticels in potato tubers. **(A)** Lenticel density (number of lenticels per surface area) per section (stolon, lateral, and apical) of four potato cultivars (‘VR808’, ‘Saturna’, ‘Hermes’, and ‘Lady Claire’) stored at 10 °C for 5 months or until sprouting. The error bar represents the LSD_0.05_ for cultivar*tuber position interaction, which was 1.4 lenticels cm^-2^. **(B)** Example of patterns of lenticels surrounding the apical bud (‘Lady Rosetta’) – image obtained under an optical microscope (x4 magnification).

A higher number of lenticels were found surrounding the apical bud (**Figure 2**) and these were generally three times smaller than those distributed across the skin (**Figure 2B**); the lateral and the stolon parts exhibited no discernible differences in terms of number of lenticels per cm^2^ (**Figure 2A**).

### 3.2 Density of lenticels regarding tuber size

**Figure 3:**
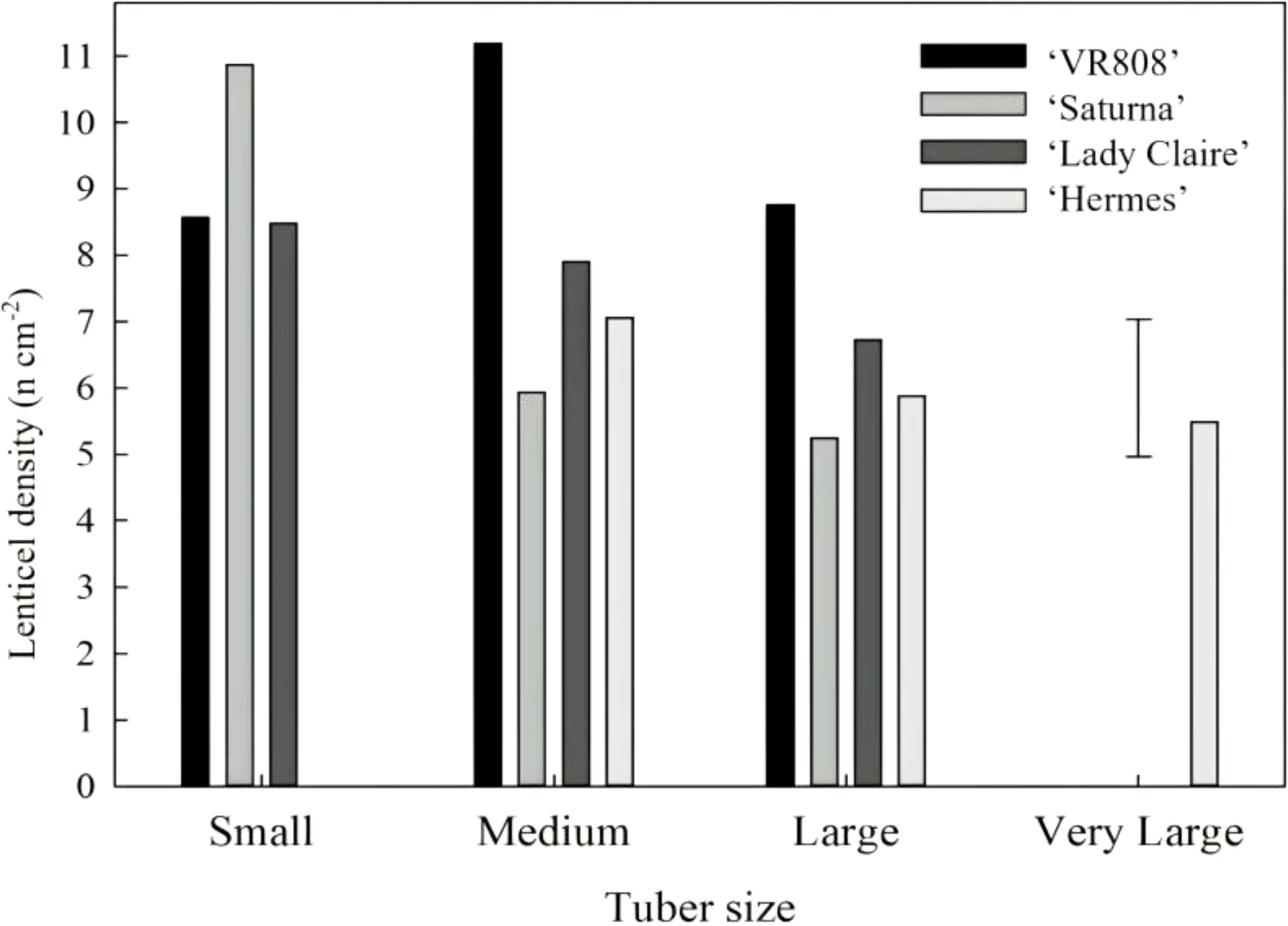
The density of lenticels according to tuber size. Tuber sizes (*x*) of cultivars ‘VR808’, ‘Saturna’, ‘Lady Claire’ and ‘Hermes’ are defined as follows: small, *x* < 80 cm^2^; medium, 80 cm^2^ < *x* < 120 cm^2^; large, 120 cm^2^ < *x* < 160 cm^2^; very large, *x* > 160 cm^2^. The error bar represents the LSD_0.05_ for cultivar*tuber size interaction (n=6 replicates of 6 tubers per replicate in each size group and cultivar), which was 2.16.

The density of lenticels was not affected by tuber size within each cultivar, since it remained cultivar-dependent. ‘Hermes’, ‘Lady Claire’, and ‘VR808’ cultivars exhibited differences among them but had steady density of lenticels regardless of tuber size. In contrast, ‘Saturna’ followed a completely different trend: small tubers showed double the density of lenticels (12 lenticels cm^-2^) than medium and large tubers (**Figure 3**).

### 3.3 Gas exchange - respiration rate

**Figure 4:**
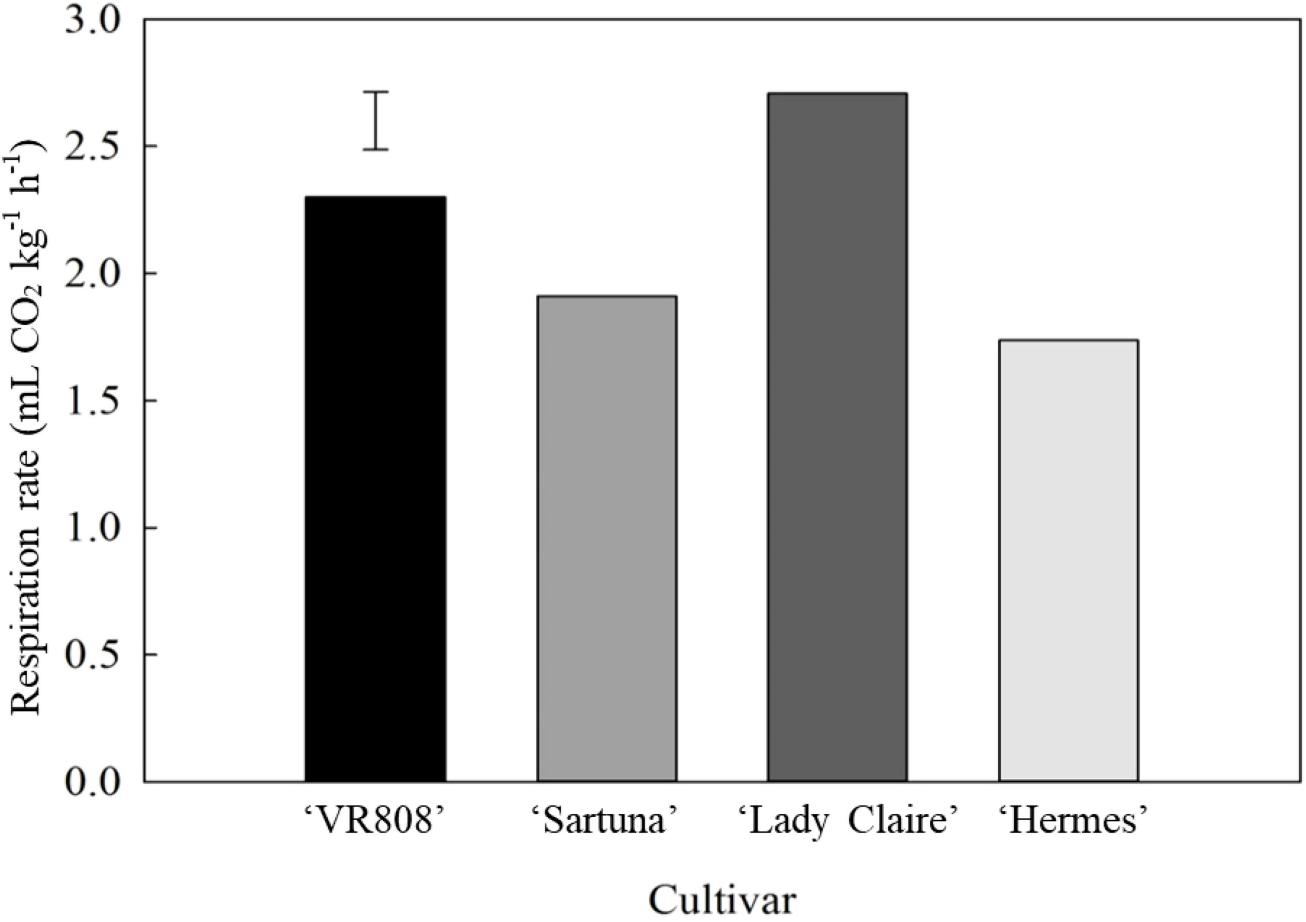
Respiration rate (mL CO_2_ kg^-1^ h^-1^) of potato tubers. Potato cultivars ‘VR808’, ‘Saturna’, ‘Lady Claire’, and ‘Hermes’ were stored for five months at 10 °C, and the measurements were carried out at 20 °C (n=6). The error bar represents the LSD_0.05_ for cultivar*tuber position interaction, which was 0.31.

‘Hermes’, with the largest tubers, showed the lowest respiration rate, which was 1.5-fold lower than that of ‘Lady Claire’. However, significant differences in the respiration rate were observed among the tested cultivars of the same size category (**Supplementary Table 1**), where ‘Lady Claire’ had higher respiration rate (2.75 and mL CO_2_ kg^-1^ h^-1^) than ‘Saturna’ (1.9 mL CO_2_ kg^-1^ h^-1^) (**Figure 4**). Consequently, ‘Saturna’ and ‘Lady Claire’ were selected for additional analyses of respiration and ethylene efflux.

**Figure 5:**
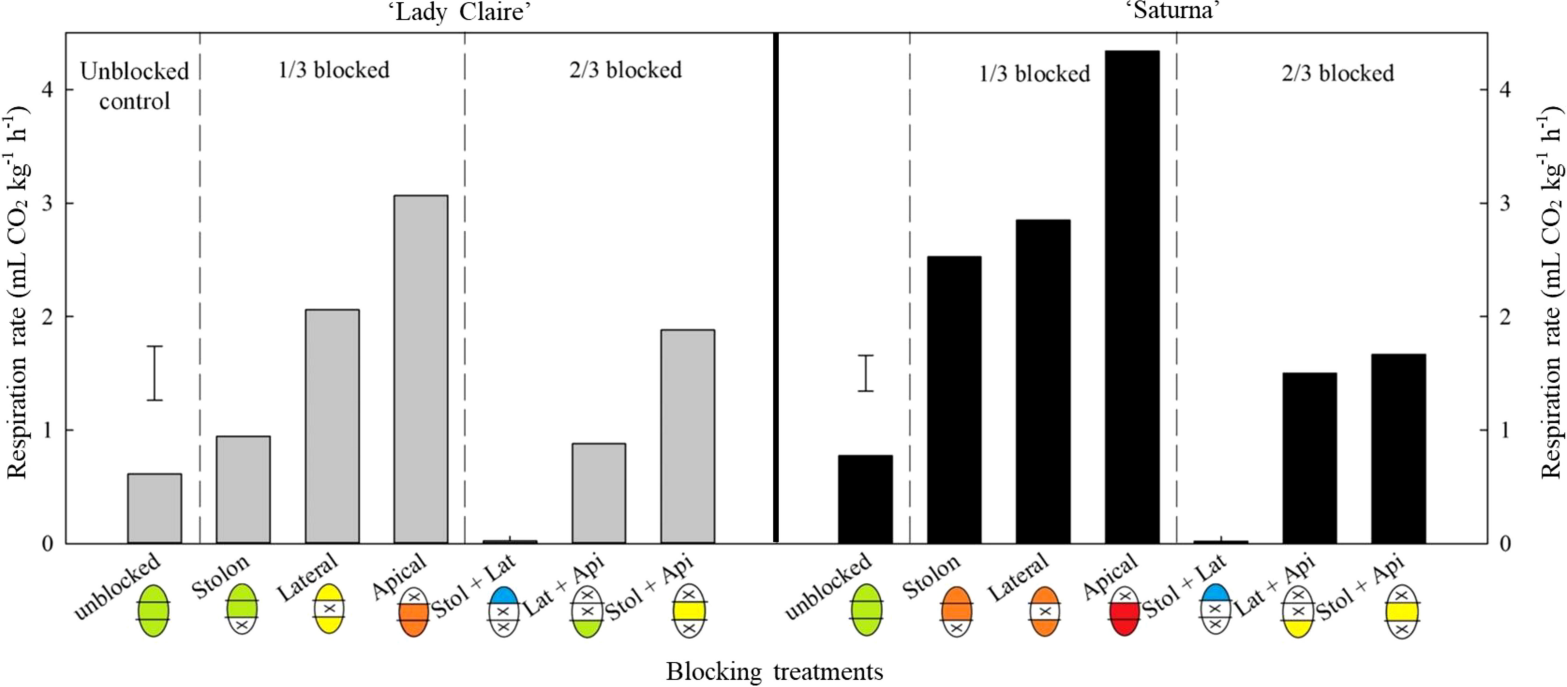
Respiration rate of potato tubers per blocking combination (‘Lady Claire’ and ‘Saturna’). The average respiration rate of ‘Lady Claire’ and ‘Saturna’ tubers (five tubers per blocking combination) is expressed as CO_2_ production (mL CO_2_ kg^-1^ h^-1^). The ‘X’ indicates the blocked section (*i.e.* stolon, lateral, apical). Colour scale of the potato schematics along the x-axis shows the overall respiration rate levels from low to high: blue, up to 0.5 < green, up to 1.5 < yellow, up to 2.5 < orange, up to 3.5 < red, over 3.5 mL CO_2_ kg^-1^ h^-1^. The dashed black vertical lines divide the plots, from left to right: unblocked tubers, a third of the blocked tuber surface (1/3), and 2/3 of the blocked tuber surface. The error bars represent the LSD_0.05_ values for the blocking treatments, which were 0.64 for ‘Lady Claire’ and 0.33 for ‘Saturna’.

Blocking lenticels in individual sections (each representing one-third of the tuber surface area, **Supplementary Figure 3**), resulted in increased respiration rates in both cultivars (**Figure 5**). When apical lenticels were blocked, respiration rate increased five-fold, whereas it decreased 30-fold when the apical section remained the only unblocked pathway for gas exchange.

In ‘Saturna’, lateral and stolon lenticels contributed equally to respiration when they were the only functional gas exchange pathways (with two-thirds of the surface area blocked). Similarly, when these sections were individually blocked, respiration rate increased 1.7 times. In contrast, ‘Lady Claire’ exhibited a different response, where lateral lenticels increased respiration by two-fold compared to stolon lenticels (with two-thirds of the surface area blocked) and to unblocked tubers. However, this higher contribution of lateral lenticels was not consistently observed when only one-third of the surface was blocked. Unexpectedly, respiration was approximately 2-times lower when the stolon section lenticels were blocked compared to when the lateral section lenticels were blocked (**Figure 5**).

### 3.4 Morphological changes in lenticels during storage

The morphology of lenticels varied across the cultivars (‘Lady Claire’, ‘VR808’ and ‘Lady Rosetta’), tuber sections (apical, lateral, and stolon), and treatments (+/-CIPC and +/- 24 h ethylene). Notably, ‘Lady Rosetta’ sprouted earlier, with storage (10 °C) terminated after 45 days, while the other two cultivars lasted 199 days. The percentage of erupted lenticels (**Supplementary Figure 4**) reached the highest values between bud movement and sprouting in all varieties, regardless of the treatment and tuber sections evaluated (**Table 1**).

**Table 1:** Respiration rate of whole tubers and percentage of erupted lenticels of apical and lateral sections in ‘Lady Rosetta’.

	After 17 days of storage			After 45 days of storage		
Treatments	Log_10__RR (mL kg^-1^ h^-1^)	Lateral erupted lenticels (%)	Apical erupted lenticels (%)	Log_10__RR (mL kg^-1^ h^-1^)	Lateral erupted lenticels (%)	Apical erupted lenticels (%)
- CIPC/- Et	1.00 ± 0.04	66.72 ± 7.01	48.81 ± 7.30	2.61 ± 0.18	83.12 ± 3.08	81.27 ± 1.65
+CIPC/- Et	1.16 ± 0.09	81.44 ± 6.27	47.08 ± 8.80	2.68 ± 0.12	77.31 ± 2.94	79.10 ± 1.39
-CIPC/+ Et	1.33 ± 0.11	78.52 ± 5.25	81.38 ± 3.67	2.78 ± 0.12	76.59 ± 1.92	79.78 ± 2.63
+CIPC/+Et	0.93 ± 0.20	55.06 ± 12.23	59.67 ± 13.96	2.85 ± 0.13	82.31 ± 2.31	79.95 ± 2.52

Values represent mean ± standard error. Control tubers (no CIPC nor ethylene treated [-CIPC/-Et]); CIPC alone treated tubers (+CIPC/-Et); ethylene alone treated tubers (-CIPC/+Et); CIPC and ethylene treated tubers (+CIPC/+Et).

Respiration rate was measured at ambient temperature after 17 days (bud movement stage) and 45 days (sprouting stage) of storage at 10 °C. The respiration rate as CO_2_ production (mL kg^-1^ h^-1^) was log-transformed (Log_10_ (RR)) to meet ANOVA requirements.

**Figure 6:**
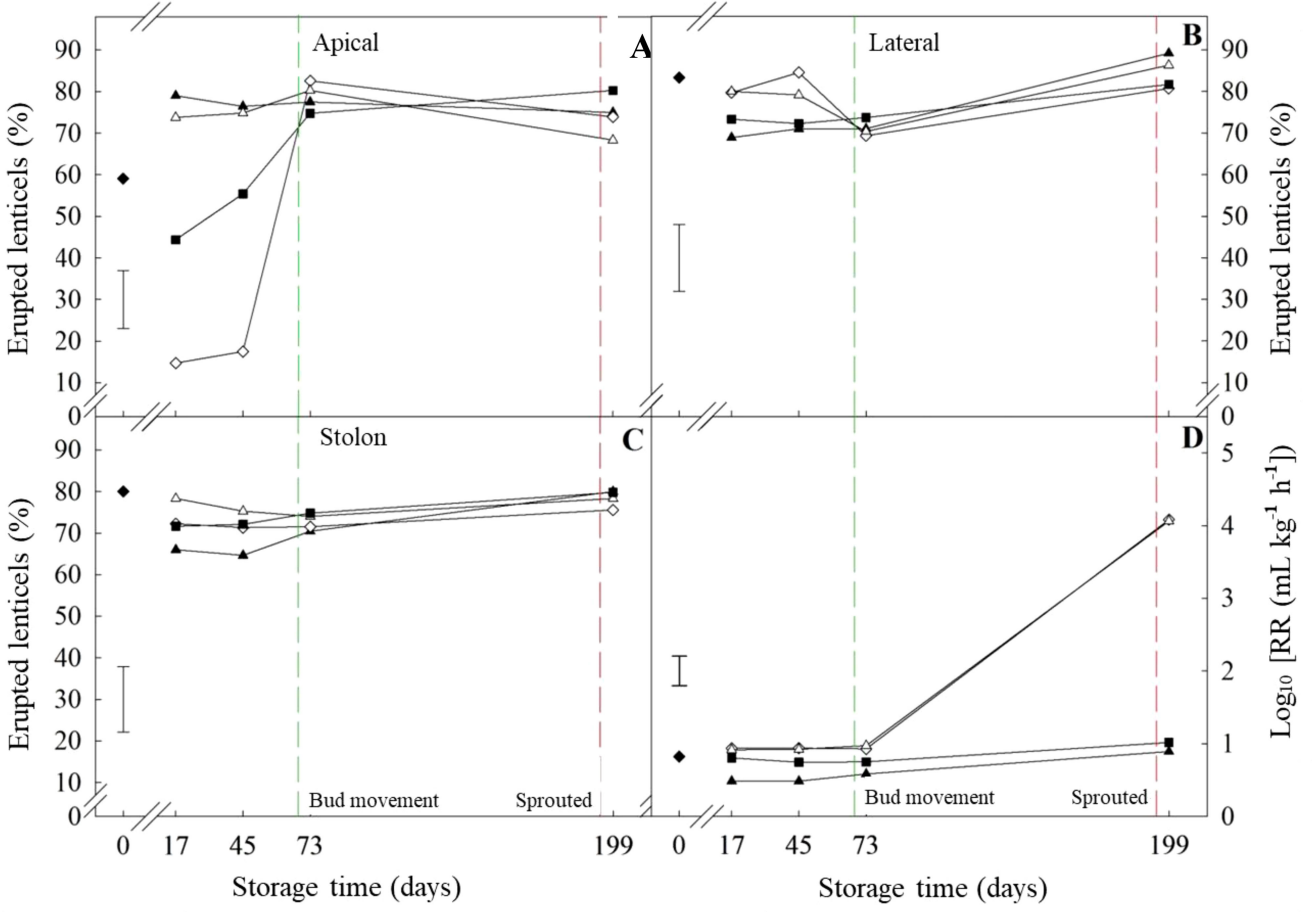
Erupted lenticels and respiration rate of ‘VR808’ potato tubers Percentage of erupted lenticles in the apical **(A)**, lateral **(B)**, and stolon **(C)** sections has been shown. Tubers underwent four treatments at 10 °C: (■) Untreated; (▲) CIPC-treated; (◊) ethylene-treated; (Δ) CIPC- and ethylene-treated. (♦) shows values at time zero. The green and red dash lines show bud movement and sprouted tubers, respectively. **(D)** Respiration rate as CO_2_ production (mL kg^-1^ h^-1^) of tubers under previous treatments was log-transformed (Log_10_ (RR)) to meet ANOVA requirements. The error bars represent the LSD_0.05_ values for treatment*storage time interactions, which were 14.4, 13.1, 16.8, and 0.29 for erupted apical lenticels **(A)**, erupted lateral lenticels **(B)**, erupted stolon lenticels **(C)**, and respiration rate, respectively.

Control tubers (those not treated with either CIPC or ethylene) of ‘Lady Rosetta’ had more erupted lenticels in the lateral section (66.72 ± 7.01) compared to the apical section (48.81 ± 7.3) at 17 days of storage (bud movement stage). However, both sections had over 80% of erupted lenticels when sprouted (**Table 1**). Ethylene alone induced the eruption of lenticels in the apical section at 17 days and led to a significant increase in respiration rate (1.33-fold higher than the untreated tubers). At that time point, CIPC downregulated the ethylene-induced effect on both lenticel eruption and respiration (**Figure 6**).

The percentage of erupted lenticels was *circa* 80% at the time of bud movement (*circa* 70 days of storage) in every section of both long-term storage cultivars (‘Lady Claire’ and ‘VR808’) and regardless of treatments (-/-; +/-; -/+; +/+, CIPC and ethylene, respectively). Before bud movement, ‘VR808’ showed fewer variations than ‘Lady Claire’ in lenticel eruption following treatments. In ‘VR808’, lateral and stolon sections had a high percentage of erupted lenticels (*circa* 80%) during storage, irrespective of treatment (**Figures 6B, C**). Additionally, a high percentage of erupted lenticels was found in the apical section of CIPC-treated tubers throughout the storage period. Similar to ‘Lady Rosetta’, CIPC appeared to reduce the ethylene-induced suberization of lenticels. The non-CIPC treated tubers significantly varied with ethylene treatment; the lowest percentage of erupted lenticels was observed in tubers treated with ethylene alone (-CIPC/+Et) (*circa* 14%), while control tubers (-CIPC/-Et) showed an intermediate value (*circa* 49%) (**Figure 6**).

**Figure 7:**
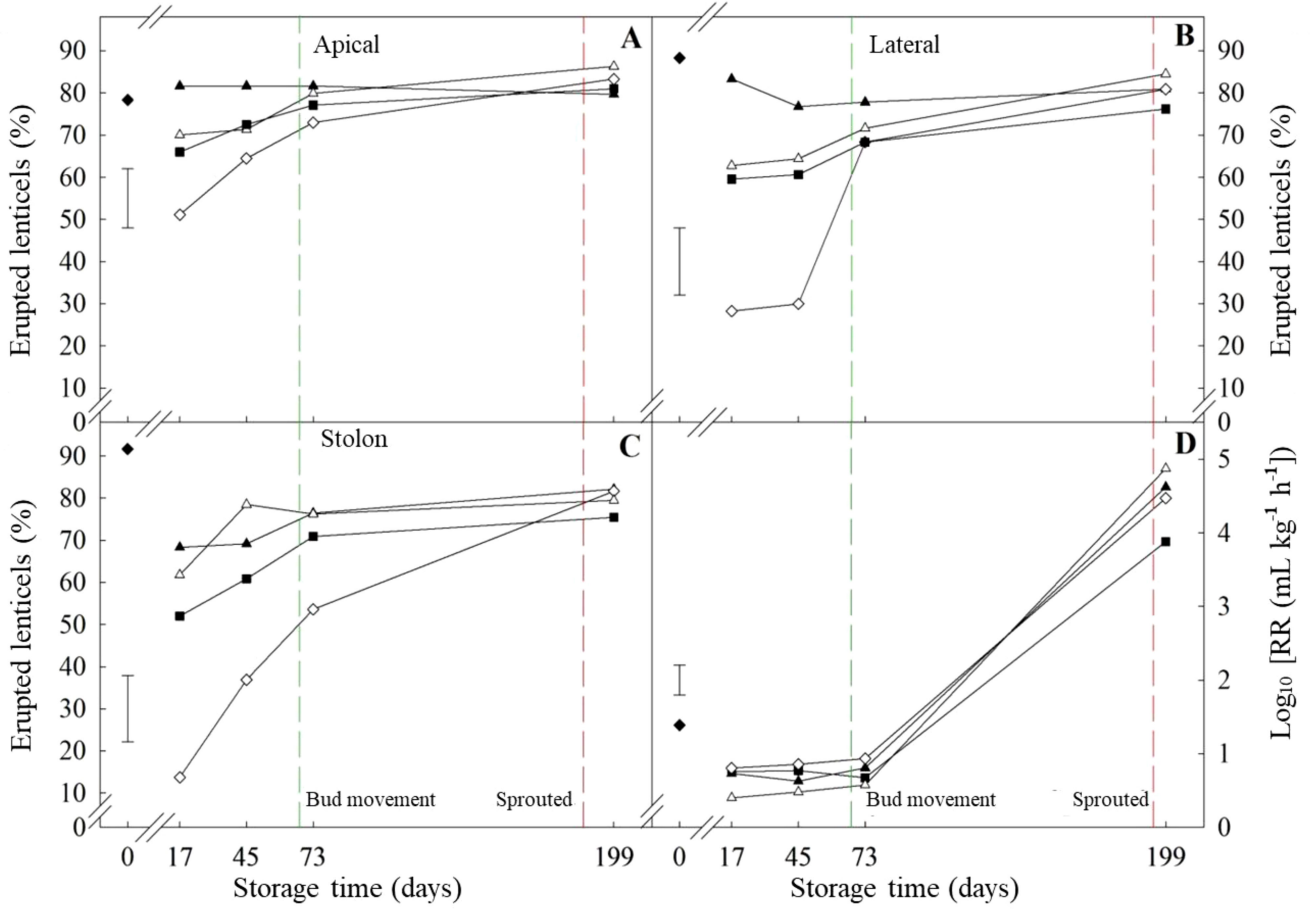
Erupted lenticels and respiration rate of ‘Lady Claire’ potato tubers. Percentage of erupted lenticels in the apical **(A)**, lateral **(B)**, and stolon **(C)** sections has been shown. Tubers underwent four treatments at 10 °C: (■) Untreated; (▲) CIPC treated; (◊) ethylene treated; (Δ) CIPC and ethylene treated. (♦) shows values at time zero. The green and red dash lines show bud movement and sprouted tubers, respectively. **(D)** Respiration rate as CO_2_ production (mL kg^-1^ h^-1^) of tubers under previous treatments was log-transformed (Log_10_ (RR)) to meet ANOVA requirements. The error bars represent the LSD_0.05_ values for treatment*storage time interactions, which were 15.2, 16.8, 16.3, and 0.44 for erupted apical lenticels **(A)**, erupted lateral lenticels **(B)**, erupted stolon lenticels **(C)**, and respiration rate, respectively.

The response to ethylene treatment alone was the main difference between cultivars prior to bud movement. ‘VR808’ was affected only in the apical section while the whole tuber of ‘Lady Claire’ responded to ethylene alone. The apical section of ‘Lady Claire’ showed analogous trends to ‘VR808’, but with initially higher percentages at day 17 (-CIPC/+Et *circa* 50%). Meanwhile, the initial percentages in the stolon were similar to ‘VR808’, yet the ethylene alone treatment displayed a gradual increase until day 73 (after bud movement) (**Figures 7A–C**). ‘Lady Claire’ exhibited a 4-fold increase in the respiration rate between bud movement and sprouting (**Figure 7D**), with variations independent of treatments. Conversely, ‘VR808’ experienced a 4-fold increase in respiration rate only following ethylene treatment, while CIPC did not enhance respiration at sprouting (**Figure 6D**).

### 3.5 Characterization of lenticels

**Figure 8:**
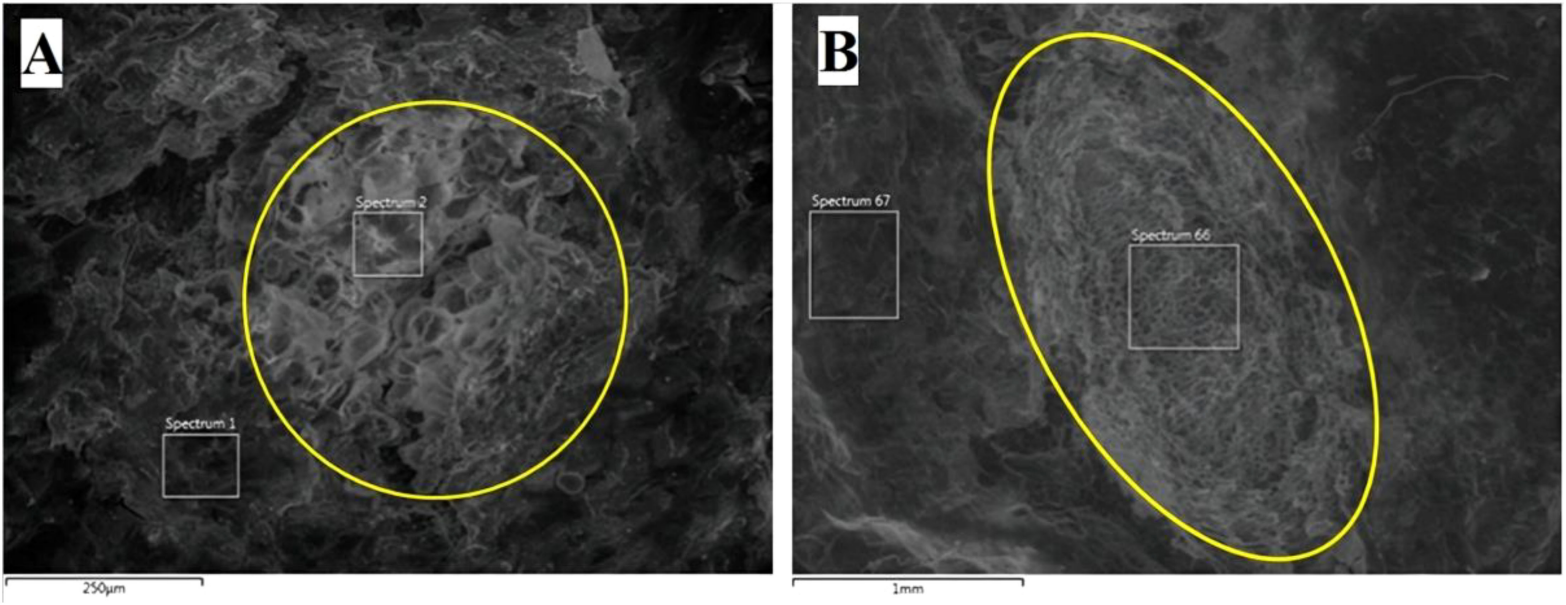
Environmental Scanning Electron Microscope (ESEM) images of ‘VR808’ lenticels. Potatoes were stored at 10 °C for 199 days. **(A)** ESEM image of a lateral lenticel (250 µm resolution). **(B)** Potato tuber stolon lenticel with an elongated shape (1 mm resolution).

Clear differences were observed in the structure and composition of cells between lenticels and skin. Lenticels were composed of globular structures, with the majority displaying a round open shape (**Figure 8A**), while some, particularly those in the stolon regions, exhibited a more elongated shape (**Figure 8B**). Notably, lenticels in the stolon section displayed a distinct structure compared to those in the lateral and apical regions, featuring internal polygonal cells similar to skin cells. The resolution of the images showed the differences in lenticels size. Lenticels at the stolon and lateral sections ranged from 1 mm (**Figure 8B**) to 100 µm (**Figure 8A**), while the apical lenticels ranged from 500 to 50 µm.

**Figure 9:**
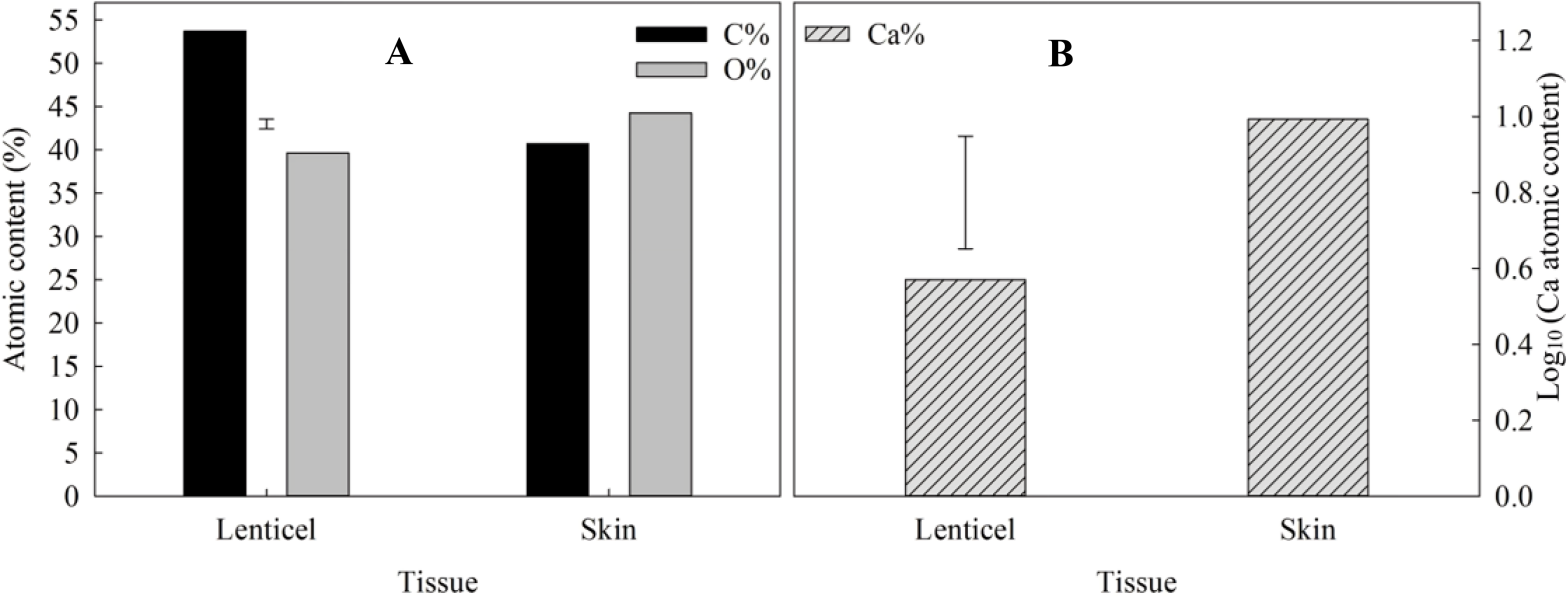
Atomic composition in lenticles and skin of CIPC-treated ‘VR808’ potato tubers. **(A)** Atomic content (%) of carbon (C%) and oxygen (O%) in lenticels and skin of CIPC-treated potato tubers. **(B)** Logarithm of atomic content of calcium (Log_10_ (Ca%)) in lenticels and skin of CIPC-treated potato tubers. The error bars represent the LSD_0.05_ values for different tissues, which were 1.94 for atomic O and C **(A)** and 0.26 for Ca **(B)**.

The atomic composition and abundance of elements carbon (C), and oxygen (O) are shown in **Figure 9A**. Statistical analysis revealed significant differences in the percentages of carbon and oxygen between lenticels and skin. Specifically, lenticels exhibited a higher carbon content (59.57%) compared to skin (47.95%), whereas the oxygen content was higher in skin (43.56%) than in lenticels (36.69%). Additionally, the analysis showed variations in calcium content, with skin containing higher levels (0.993%) compared to lenticels (0.571%) (**Figure 9B**).

## 4 Discussion

### 4.1 Tuber lenticel density is partially genotype-dependent

The density of lenticels of ‘Hermes’, ‘Lady Claire’, and ‘VR808’ remained consistent regardless of tuber size, which was in agreement with findings by Scott et al. (1996). In contrast, ‘Saturna’ demonstrated a distinct pattern; small tubers showed double the density of lenticels (12 lenticels cm^-2^) compared to medium and large tubers (**Figure 3**). This inverse relationship between tuber size and lenticel density in this cultivar suggests that lenticel density is not solely determined by tuber size within varieties but is largely dependent on cultivar. Given the intrinsic role of tuber gas exchange in postharvest physiology, it is crucial to identify or characterize genotypes with optimal gas exchange properties to enhance the effectiveness of postharvest technologies such as controlled atmosphere (CA) storage.

The results we found for ‘Saturna’ tubers are somehow supported by the study published about lenticel formation in ‘Gala’ apples by Turketti et al. (2014). Comparing ‘Gala’ apples at different development stages, the authors found that the density of lenticels decreased with the increase in fruit size. This may be explained by genetic control of lenticel formation occurring prior to tuber enlargement. Lenticels did not form in growing nodules of *Lotus japonicus* when treated with an auxin inhibitor (Takanashi et al., 2011); this seemed to confirm that lenticel formation may occur mainly during early developmental stages in different species (Turketti et al., 2014; Khanal et al., 2020; Kritzinger and Lötze, 2019). In tuber and root crops, lenticels, initially composed of radial files of undifferentiated cells, expand by cell division in the phellem layer, forming intercellular spaces that rupture the epidermis/periderm, facilitating gas exchange, an adaptation to hypoxia (Tyner et al., 1997; Bethke, 2023).

The role of phytohormones, particularly auxins and cytokinins, has been well-characterized in potato tubers during tuberization and lenticel development (Roumeliotis et al., 2012; Bethke, 2023). Specifically, auxin and cytokinin signaling pathways regulate cell division leading to lenticel proliferation (Clark et al., 2013). A similar mechanism has been observed in *Salix viminalis*, where hypoxia tolerance was linked to auxin-regulated lenticel formation (Jackson and Attwood, 1996). ‘Saturna’ data suggested that lenticel formation is likely to occur prior to tuber enlargement; however, it cannot be confirmed since the density of lenticels was independent of tuber size for the rest of the cultivars (‘VR808’, ‘Lady Claire’ and ‘Hermes’) (**Figure 3**). The differences in size between stolon lenticels and those in other regions may suggest that they are formed earlier during tuberization. Further investigation into the significance of these differences is warranted, as they may indicate unique functionality for stolon lenticels compared to those in other areas of the tuber.

### 4.2 Lenticel size and spatial distribution may impact gaseous exchange and therefore senescence-related respiration of potato tubers

‘Hermes,’ which had the largest tubers, exhibited a respiration rate 1.5 times lower than that of ‘Lady Claire,’ likely due to its lower lenticel density (**Figure 3**). The five-fold increase in respiration rate observed when apical lenticels were blocked, and the 30-fold decrease when the apical section was the only unblocked area in ‘Lady Claire’ and ‘Saturna’ cultivars, suggest a stress-induced physiological response triggered by blocking the apical section, with the highest lenticel density.

The results obtained in the current study on potatoes showed spatial differences in lenticel abundance and size; they were more numerous and smaller size in the apical section (**Figure 2A**) compared to lateral and stolon lenticels. Lenticels are key sites for gas exchange (O_2_, CO_2_, and water vapor), playing a vital role in senescence-related respiration and heat dissipation (Tyner et al., 1997). To delay senescence and prolong postharvest-life, ongoing research investigates the respiratory metabolism of fresh produce through CA storage, achieved by reducing O_2_ and increasing CO_2_ partial pressures and slowing down respiration in low ethylene-producing crops. We hypothesize that lenticel number and density primarily impact gas permeability, with excessive open lenticels undesirably increasing gas exchange/diffusion and moisture loss, while lower number of lenticels could cause anoxia or hypoxia under low oxygen CA conditions. These results highlighted for the first time that the position of lenticels along the tuber differentially may mediate respiration (*i.e.* apical contribution was negligible in both cultivars).

### 4.3 Lenticel eruption and lenticel morphology could be used as biomarkers of dormancy break

The percentage of erupted lenticels reached the highest values between bud movement and sprouting in all varieties (**Table 1**; **Figures 6**, **7**), regardless of the treatment and tuber sections surveyed. This might be explained by speculating about the activation of different metabolic pathways (*i.e.* non-structural carbohydrate metabolism, respiration, and plant growth regulators) supporting the differentiation of meristematic cells and sprout growth. The increase in respiration rate coincided with the onset of sprouting in all cultivars, even if few differences were found between treatments (**Table 1**; **Figure 6**). Both the lateral and apical sections had over 80% of erupted lenticels upon sprouting, supporting the hypothesis that cell division activation is involved in dormancy break. This finding suggests that postharvest lenticel eruption could serve as a potential marker for dormancy break. Ethylene alone induced the eruption of lenticels in the apical section at 17 days and led to a significant increase in respiration than the untreated tubers (**Table 1**). At that time, CIPC seemed to reduce the ethylene-induced effect on both lenticel eruption and respiration. CIPC inhibits meristematic cell division (Paul et al., 2016) and it may also counteract the eruption of lenticels which is a consequence of cell divisions underneath the suberin layer (**Figure 6**).

These results confirmed previous findings which describe that the efficacy of ethylene is related to the cultivar. For example, Foukaraki et al. (2014) reported different ethylene-related responses on sprout suppression and accumulation of reducing sugars in several potato cultivars. Ethylene treatment alone (-CIPC/+Et) showed opposite effects on the apical lenticels between short- (‘Lady Rosetta’) and long-storage cultivars (‘VR808’ and ‘Lady Claire’). Comparing the phenological stage of tubers was challenging, as ‘Lady Rosetta’ demonstrated advanced bud movement from the first sampling point. Short-term (24 h) ethylene treatment seemed to induce lenticel suberization prior to bud movement in ‘Lady Claire’ and ‘VR808’, while it promoted the eruption of lenticels at dormancy break. Moreover, the data showed erupted lenticels before bud movement following CIPC treatment which suggests that CIPC inhibited suberization. Furthermore, CIPC was able to counteract the ethylene-induced suberization. This may relate to CIPC action inhibiting cell division (Briddon, 2006; Paul et al., 2016). A similar trend was observed in sweetpotato roots (*Ipomoea batatas* L.), where exogenous ethylene application during defoliation promoted lenticel proliferation, while treatment with the ethylene antagonist 1-MCP inhibited it (Villordon, 2012; Clark et al., 2013). This suggests that ethylene plays a key role in signaling and regulating the physiological mechanisms underlying lenticel formation as a morphological adaptation to stress.

The highest amounts of erupted lenticels appeared after bud movement (regardless of treatments); despite that, the respiration rates showed significant differences only at sprouting. The metabolic activation at dormancy break may promote lenticels to erupt. This morphological change would support the emission of CO_2_ resulting from elevated respiration, which provides the energy required for sprout growth. High levels of CO_2_ following ethylene treatments were reported by Briddon (2006) and Bethke (2014) found that short-term exposure to high doses of ethylene (20–200 nL L^-1^) increased the respiration rates and enhanced differences among varieties. Similarly, Tosetti et al. (2021) observed a transient CO_2_ peak in ethylene-treated tubers (10 μL L^-1^) five days post-treatment before declining.

The variations in lenticel morphology were identified prior to bud movement, suggesting that lenticels could be used as a pre-symptomatic visualization marker of dormancy break. Respiration was not significantly affected by the variation in lenticel morphology, as it increased only at sprouting. Additionally, different varieties exhibited varying sensitivity to the tested treatments. Consequently, establishing a direct link between respiration and morphological changes in lenticels remains challenging.

### 4.4 Digital imaging has potential as a high-resolution lenticel phenotyping method

Reliable high-resolution phenotyping methods for tuber lenticels based on digital imaging and ESEM (**Figure 8**) coupled with image analyses using ImageJ software were developed (**Supplementary Figure 2**). ESEM appears to be a promising tool for studying potato lenticels and their functionality. Image analysis enabled accurate identification and counting of lenticels, including those smaller than 0.05 cm in diameter, enhancing counting precision beyond previous reports that only documented lenticels above 0.05 cm (Burton, 1950, 1965; Wigginton, 1973). This study represents the first application of digital imaging as an innovative high-resolution tool for lenticel phenotyping, providing a foundation for more objective and detailed lenticel trait characterization. However, the reliance on digital imaging and ESEM on manual image processing makes them laborious for large-scale studies. Future research should focus on developing novel high-throughput, non-destructive technologies such as machine vision (Bhargava and Bansal, 2021), hyperspectral imaging (Song et al., 2024), and optical coherence tomography (Li et al., 2019), integrated with machine learning, to digitally phenotype lenticel density and spatial distribution.

Mapping of calcium using X-ray microanalysis revealed clusters predominantly located in the inner layers of the periderm (**Supplementary Figure 5**). This analysis supports the hypothesis of structural differences between lenticels and potato skin, which may directly influence their functionality. The observed lower abundance of calcium within lenticels compared to the skin (**Figure 9**) suggests a potential correlation with lenticel eruption. Given the role of calcium in cell wall integrity (Subramanian et al., 2011) and intracellular adhesion (Ginzberg et al., 2012), a reduction in its content could indicate the initiation of lenticel eruption. This novel phenotyping of lenticels at such detailed resolution enhances our understanding of their anatomical and structural differences along the tuber. The successful application of the ESEM technique in analyzing lenticels and skin atomic composition underscores its potential for further investigation. Additionally, future studies could explore the role of calcium in lenticels, given their importance as primary pores for transpiration and respiration.

## 5 Conclusions

This study provides novel insights into the role of lenticels in potato tuber gas exchange and dormancy regulation during postharvest storage. Our findings reveal that lenticel density, morphology, and spatial distribution vary among cultivars, influencing respiration rates and potentially serving as early indicators of dormancy break. The results highlight that the apical sections, which exhibit the highest lenticel density, are associated with elevated respiration rates as a stress-induced physiological response upon blockage, compared to the lateral and stolon sections. The observed changes in lenticel morphology, particularly the eruption of lenticels preceding bud movement, suggest that lenticel eruption may serve as a pre-symptomatic marker for dormancy break. Additionally, the study underscores the potential for optimizing CA storage strategies by leveraging the knowledge of the lenticel-mediated gas exchange. The differential responses of cultivars to ethylene and CIPC treatments further emphasize the need for cultivar-specific storage protocols to maximize sprout suppression and minimize postharvest losses. However, ethylene effect on lenticel morphology remained unclear, inducing suberization during early storage but promoting eruption near bud movement. Additionally, there was no concurrent increase in respiration rate with a higher number of erupted lenticels, suggesting that lenticels may have additional, yet unknown, functions. Future research should explore the genetic and molecular mechanisms regulating lenticel development and function in potato tubers. Additionally, the integration of high-throughput imaging technologies and machine learning approaches could enhance characterization of lenticel properties, facilitating the development of targeted storage solutions. By advancing our understanding of lenticel physiology, this study contributes to the broader goal of improving postharvest management strategies for potatoes and other tuberous and root crops.

## Data Availability

The original contributions presented in the study are included in the article/**Supplementary Material**. Further inquiries can be directed to the corresponding author/s.
