# Supplementary material for: Morphological changes of lenticels and their role in gas exchange and sprouting physiology of potato tubers during postharvest storage
Source: Front Plant Sci. 2025 Jun 11;16:1595828. doi: 10.3389/fpls.2025.1595828 (PMC12188445; doi:10.3389/fpls.2025.1595828)
Supplement: Supplementary file 1 [file DataSheet1.docx]

Supplementary Material

Morphological Changes of Lenticels and their Role in Gas Exchange and Sprouting Physiology of Potato Tubers During Postharvest Storage

Lembe S. Magwaza^1^, Antonio J.B. Bernal^1^, M. Carmen Alamar^1*^, Leon A. Terry^1^

^1^ Postharvest Research Group, Cranfield University, Bedfordshire, MK43 0AL, United Kingdom

*** Correspondence:**M. Carmen Alamar
[m.d.alamargavidia@cranfield.ac.uk](mailto:m.d.alamargavidia@cranfield.ac.uk)

# Supplementary Tables

Supplementary Table 1. Mean surface area (cm^2^) of six tubers for each cultivar.

|  |  | **Cultivar** | |  |
| --- | --- | --- | --- | --- |
| **Size** | **‘VR808’** | **‘Saturna’** | **‘Hermes’** | **‘Lady Claire’** |
| Small | 61.3 ± 1.30 | 66.0 ± 1.90 |  | 67.2 ± 1.60 |
| Medium | 91.2 ± 2.45 | 107.3 ± 2.59 | 94.5 ± 3.84 | 84.0 ± 1.58 |
| Large | 139.4 ± 3.88 | 130.6 ± 4.77 | 117.5 ± 4.12 | 139.1 ± 4.86 |
| Very large |  |  | 195.1 ± 6.12 |  |

Values represent mean ± standard error.

# Supplementary Figures


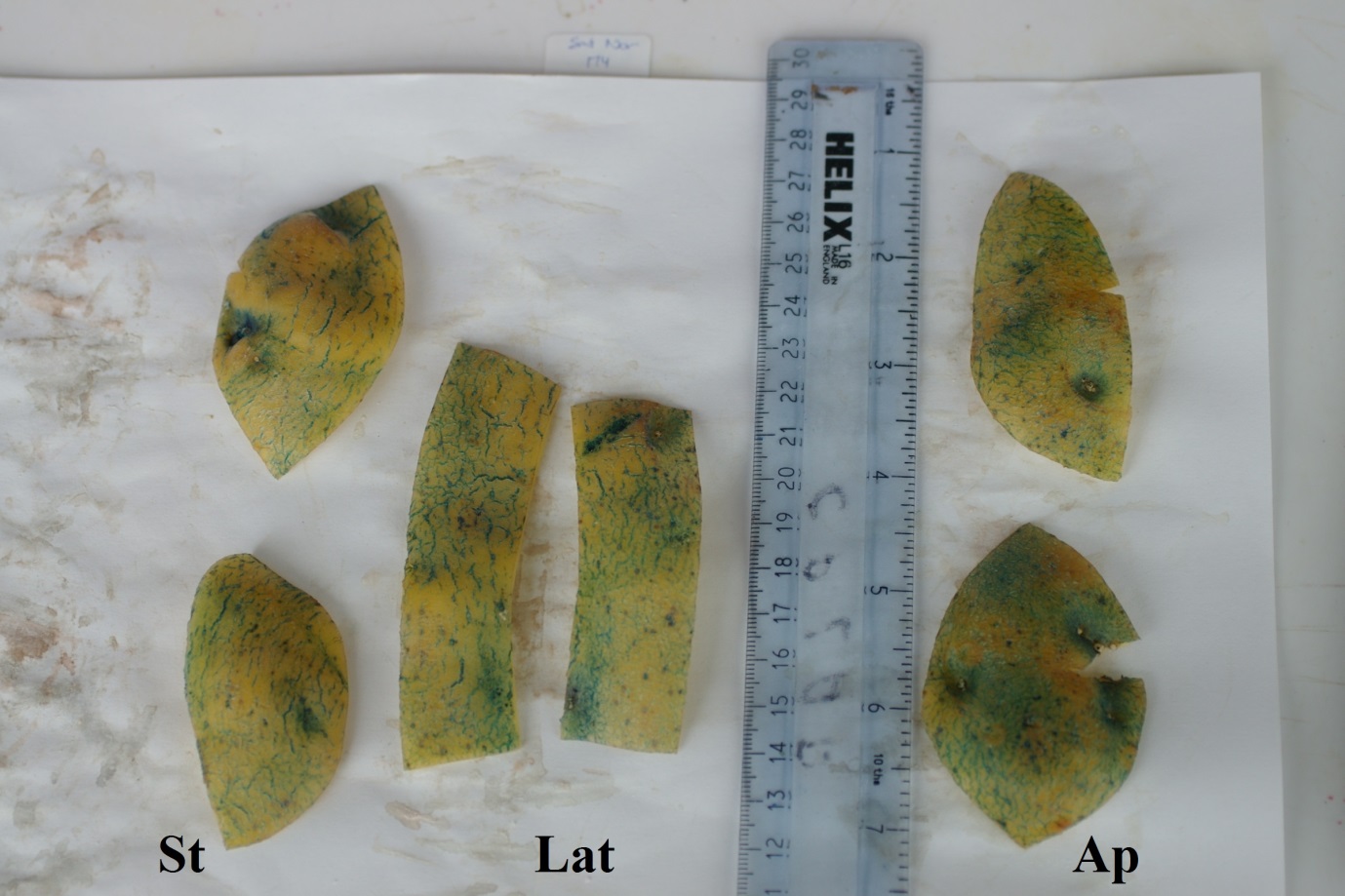


Supplementary Figure 1. An example of a potato skin sample after submerging in a methylene blue dye solution (1.5 g L^-1^) for 15 minutes. The sample was divided into three sections: Stolon (St); Lateral (Lat); and Apical (Ap) in preparation for image analysis.


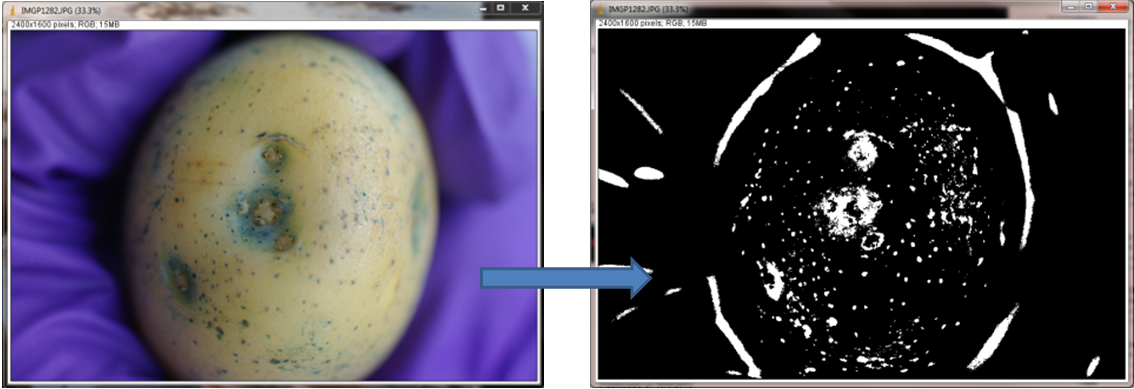


**A**


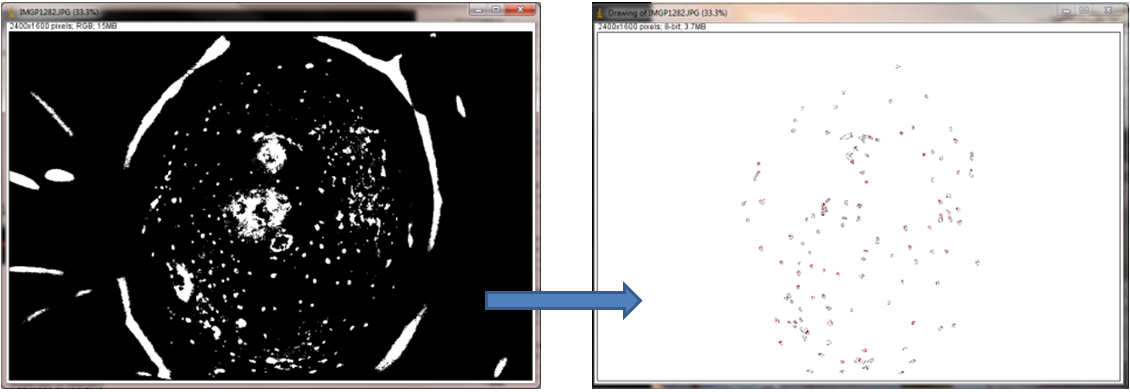


**B**

**Supplementary Figure 2**. Image analysis steps using ImageJ software. **A)** ‘Automatic particle counting’, the threshold setup of the binary image, and **B)** watershed line set from the binary threshold image. ImageJ enabled accurate identification and counting of lenticels, including those smaller than 0.05 cm in diameter, enhancing counting precision beyond previous reports that only documented lenticels above 0.05 cm (Burton, 1950; Burton, 1965; Wigginton, 1973).


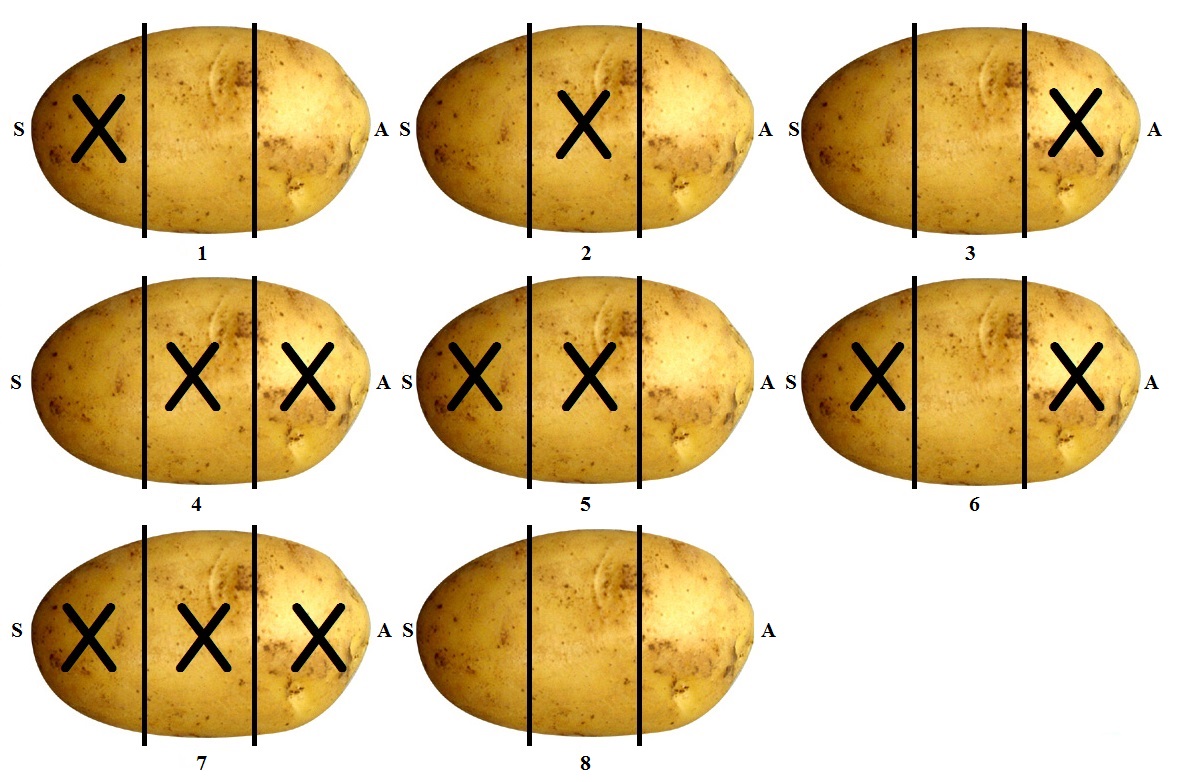


**Supplementary Figure 3.** Tuber sections (stolon [S], lateral, and apical [A]) were obstructed in eight different patterns to avoid gas diffusion through lenticels. Araldite was applied in the sections marked with an X. The Blocking patterns were numbered from 1 to 8: 1, 2, and 3 were respectively blocked at the stolon, lateral, and apical end of the tuber. Samples 4, 5, and 6 were obstructed at two positions; sample 4 at both apical and lateral positions, sample 5 at stolon and lateral positions, and sample 6 at both stolon and lateral positions. Sample 7 was obstructed in all positions, while the control was a sample left unblocked.


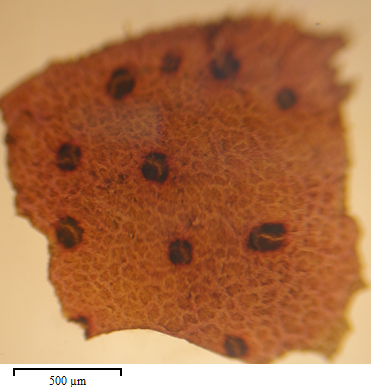

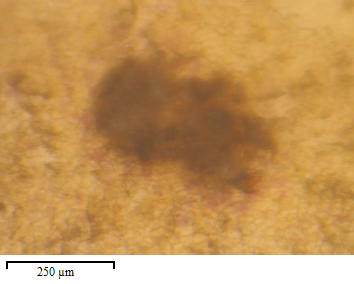


**B**

**A**

**Supplementary Figure 4.** Optical **microscopy images potato tuber skin.** Erupted lenticels found in a skin slice (2 cm^2^) of a sprouted ‘Lady Rosetta’ (**A**) and non-erupted lenticel of the ‘Lady Claire’ potato tuber (**B).** Microscopy images were respectively taken at 4X and 8X magnification.


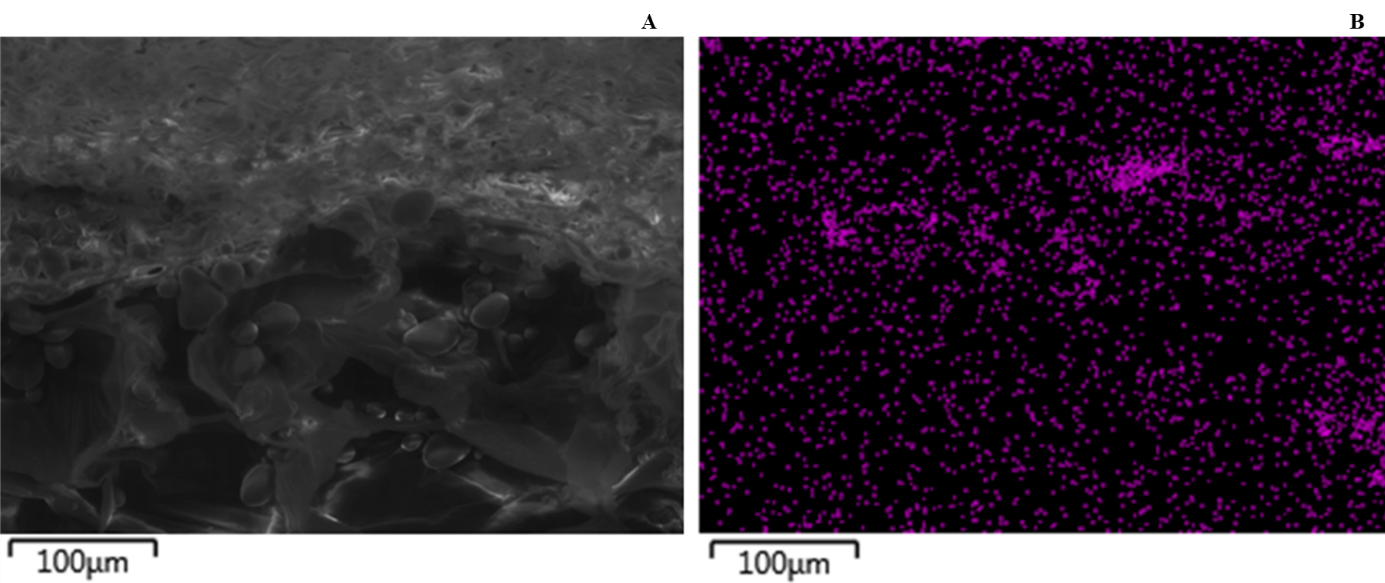


**Supplementary Figure 5.** Environmental Scanning Electron Microscope (ESEM) image of ‘VR808’ potato skin. **A:** Transversal ESEM image of potato skin obtained where periderm (upper layer) and parenchyma (lower layer) can be distinguished. Some advantages of the technique include the lack of sample preparation or metal coating requirements (Donald, 2003), and the high quality and resolution of the images obtained. **B:** Map of calcium distribution in the previous transversal image of potato skin using X-ray micro-analyser. The X-ray microanalysis represents a very interesting tool since it is possible to determine the atomic composition of samples, providing insights into the distribution of specific atoms within tissues. Despite the time-consuming nature of the mapping process from the ESEM Image, X-ray microanalysis yields precise information about the spatial distribution of elements.

References

Donald, A.M., Baker, F.S., Smith, A.C., Waldron, K.W. 2003. Fracture of plant tissues and walls as visualized by environmental scanning electron microscopy. *Ann. Bot*. 92, 73-77. <https://doi.org/10.1093/aob/mcg115>

Burton, W. G. 1950. Studies on the dormancy and sprouting of potatoes. I. The oxygen content of the potato tuber. *New Phy­tologist* 49, 121–134. [https://doi.org/10.1111/j.1469-8137.1950. tb05150.x](https://doi.org/10.1111/j.1469-8137.1950.%20tb05150.x).

Burton, W.G. 1965. The permeability of oxygen of the periderm of the potato tuber. *J. Exp. Bot*. 16, 16-23. https:// doi.org/10.1093/jxb/16.1.16.

Wigginton, M. J., 1973. Diffusion of oxygen through lenticels in potato tuber. *Potato Res.* 16, 85–87. <https://doi.org/10.1007/BF02360611>.
